# Comprehensive MR Urography Protocol: Equally Good Diagnostic Performance and Enhanced Visibility of the Upper Urinary Tract Compared to Triple-Phase CT Urography

**DOI:** 10.1371/journal.pone.0158673

**Published:** 2016-07-06

**Authors:** Mazen Sudah, Amro Masarwah, Sakari Kainulainen, Marja Pitkänen, Hanna Matikka, Vaiva Dabravolskaite, Sirpa Aaltomaa, Ritva Vanninen

**Affiliations:** 1 Department of Clinical Radiology, Kuopio University Hospital, Kuopio, Finland; 2 Institute of Clinical Medicine, University of Eastern Finland, Kuopio, Finland; 3 Department of Surgery, Urology Unit, Kuopio University Hospital, Kuopio, Finland; 4 Biocenter Kuopio and Cancer Center of Eastern Finland, University of Eastern Finland, Kuopio, Finland; University of Pécs Medical School, HUNGARY

## Abstract

**Objectives:**

To prospectively compare the diagnostic performance and the visualization of the upper urinary tract (UUT) using a comprehensive 3.0T- magnetic resonance urography (MRU) protocol versus triple-phase computed tomography urography (CTU).

**Methods:**

During the study period (January-2014 through December-2015), all consecutive patients in our tertiary university hospital scheduled by a urologist for CTU to exclude UUT malignancy were invited to participate. Diagnostic performance and visualization scores of 3.0T-MRU were compared to CTU using Wilcoxon matched-pairs test.

**Results:**

Twenty patients (39 UUT excreting units) were evaluated. 3.0T-MRU and CTU achieved equal diagnostic performances. The benign etiology of seven UUT obstructions was clarified equally with both methods. Another two urinary tract malignant tumors and one benign extraurinary tumor were detected and confirmed. Diagnostic visualization was slightly better in the intrarenal cavity areas with CTU but worsened towards distal ureter. MRU showed consistently slightly better visualization of the ureter. In the comparison, full 100% visualizations were detected in all areas in 93.6% (with 3.0T-MRU) and 87.2% (with CTU) and >75% visualization in 100% (3.0T-MRU) and 93.6% (CTU). Mean CTU effective radiation dose was 9.2 mSv.

**Conclusions:**

Comprehensive 3.0T-MRU is an accurate imaging modality achieving comparable performance with CTU; since it does not entail exposure to radiation, it has the potential to become the primary investigation technique in selected patients.

**Trial Registration:**

ClinicalTrials.gov NCT02606513

## Introduction

Recent advances in multidetector computed tomography (MDCT) technology have made possible rapid high resolution imaging in multiple phases. Research into MDCT applications has resulted in imaging protocols that produce optimal opacification, visualization and distention of the upper urinary tract (UUT) [1], CT urography (CTU) has become the primary investigation of choice for the evaluation of hematuria and high risk patients for UUT malignancy. While CTU achieves excellent pooled 96% sensitivity and 99% specificity [2], it is necessary to expose the patient to radiation which is a cause of concern and a major drawback with this technique [1].

Magnetic resonance urography (MRU) is a costly, time-consuming and expertise-demanding imaging modality. Additionally, it has also been reported to suffer from other limitations i.e. lower spatial resolution, an inability to directly visualize calcifications and a susceptibility to a wide range of artefacts [3,4]. Nevertheless, MRU has many established advantages including its high contrast resolution, good sensitivity for contrast media and most importantly, its safety. There are no fundamental limitations to utilizing MRU [5], and it is recommended in many guidelines as a suitable alternative to CTU whenever the latter is contraindicated or undesirable [6–8]. In infants and children, MRU is already a well-established imaging modality in the evaluation of the UUT [9], yet research is lacking for wider applications in adults. Previously, MRU has been shown to be highly sensitive at detecting the presence and level of an obstruction [10,11] and highly accurate in the evaluation of patients with acute flank pain [12].

The majority of MRU reports have been retrospective and based on devices with a field strength of 1.5T. In comparison to 1.5T-MRI, 3.0T imaging achieves higher signal-to-noise ratios, improved spatial resolution and faster scanning, all of which contribute to better visualization of anatomical details. The use of a higher field could theoretically mitigate some of the current limitations of MRU and help to obtain higher image resolution [3,4]. The 3.0T-MRU literature however is scant e.g. there are no comparative studies of comprehensive CTU and 3.0T-MRU protocols.

The European Society of Urogenital Radiology (ESUR) CTU Working Group defines a CTU examination as one that involves the use of MDCT with thin-slice imaging, intravenous administration of contrast medium, and imaging in the excretory phase. The ESUR guidelines list several alternative approaches to minimize the radiation dose in different clinical situations [1]. Nonetheless, a comprehensive MRU protocol must be adopted to fully evaluate the kidneys and UUT; it consists of both T2-weighted and a combination of T1-weighted sequences in different orientations and at different time intervals after the administration of contrast material to maximize the clinical value [13]. Nevertheless, there is no consensus about what represents the optimal MRU protocol.

The aim of this prospective study was to evaluate the excretory phase urothelium-surface visualization of the UUT and to compare the diagnostic performance of a comprehensive 3.0T-MRU protocol with triple-phase CTU in the evaluation of consecutive patients with hydronephrosis and in high-risk patients for UUT malignancy.

## Materials and Methods

### Study Design

This prospective study was approved by the ethics committee of Kuopio University Hospital, and written informed consent was obtained from all patients. During the study period from January-2014 through December-2015, all high-risk patients for UUT malignancy and patients with UUT hydronephrosis of unknown etiology, detected by ultrasound, and who were scheduled for triple-phase CTU, to rule out UUT malignancy, in accordance with the ESUR recommendations, were invited to participate. The exclusion criteria were: glomerular filtration rate (GFR) <45; allergy to iodinated or gadolinium contrast agents; known hypersensitivity to furosemide or sulfa; contraindications to MRU (including claustrophobia).

**Fig 1:**
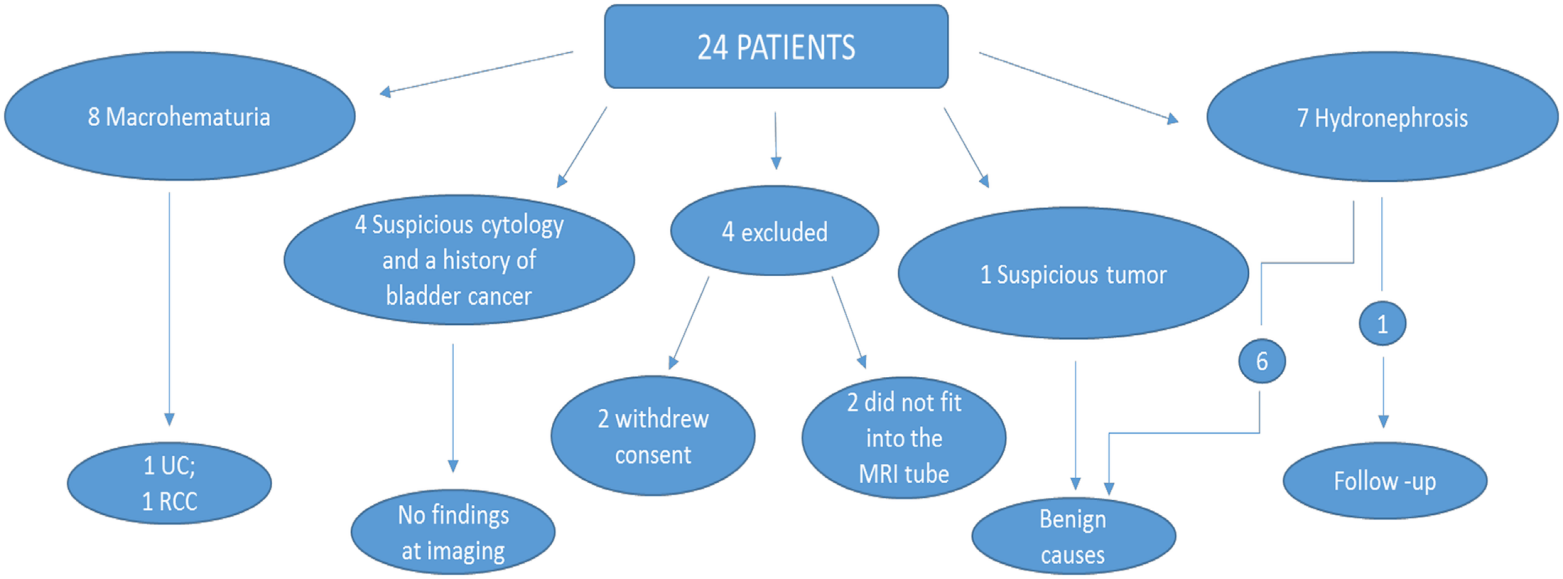
Flow chart. Flow chart of study patients, indications for imaging and results as determined by clinical evaluation, the results of imaging studies and the final histopathological diagnosis. (UC = Urothelial carcinoma; RCC = Renal Cell Carcinoma).

A total of 20 patients (12 men and 8 women, mean age 65.7 years, range 40–82 years) underwent both examinations (Fig 1). During the study period, a total of 14 other patients fulfilled inclusion criteria yet did not participate. The reference standard was CTU, and the final diagnosis was made on the basis of a combination of available clinical and imaging results and the findings of interventional examinations or procedures.

### Imaging Studies

#### MRU

3.0T-MRU protocol and sequence characteristics are described in Table 1. After T2- weighted sequences, axial T1-weighted sequence was obtained before the intravenous administration of a low-dose furosemide 0.1 mg/kg of body weight (Furesis; Orion, Espoo, Finland) to enhance diuresis (duration of activity is approximately 2 hours) with the total individual dose not exceeding 10 mg delivered 1–2 minutes before the intravenous injection of 0.05 mmol/kg gadoterate meglumine (Dotarem, Guerbet, Roissy CdG, France) via power injector (3ml/sec) followed by 20 ml of saline flush. T1-weighted sequences were obtained at different timing intervals: during the corticomedullary (30 sec; axial), nephrographic (90 sec; coronal) and excretory phases (5, 10 and 15 min; coronal and at 15 min axial). The duration of the examination was approximately 35–40 min.

**Table 1:** Magnetic Resonance (MR) and Computed Tomography (CT) Urography Imaging protocols.

**MR Urography Protocol**							
Patients were asked to void immediately before the examination; Supine position; 3.0T (Philips Achieva TX, Philips N.V., Eindhoven, The Netherlands); 16-element phased-array body coil (Sense-XL-Torso) centered over the abdomen and pelvis; Parallel imaging (acceleration factor of 2); Commercially available breath hold sequences							
Sequence	TR/TE (ms)	Flip angle	Acquisition matrix	Orientation	Slice thickness (mm)	FOV (mm)	Scanning time (s)
T2-TSE^1^	1058/80	90	224x224	axial	3.6	290x362x160	16
T2-TSE	1058/80	90	224x224	coronal	3.6	400x343x120	16
T2-TSE FS^2^	1058/80	90	224x224	axial	3.6	290x362x160	16
T2-TSE FS	1058/80	90	224x224	coronal	3.6	400x343x120	16
DWI^3^	shortest/95	90	240x240	axial	6	403x261x349	215
3D T1-TFE^4^	shortest	10	232x233	axial	4/2*	320x369x350	24
3D T1-TFE	shortest	10	232x233	Coronal	2/1*	320x350x100	16
**CT Urography protocol**							
128-slice MDCT scanner (SOMATOM Definition Edge, Siemens, Erlangen, Germany) with automatic tube-current modulation; Preparation: hydration with 1000 ml water orally 30–60 min prior to CT; voiding immediately before mounting CT table; Unenhanced CT: from the top of kidneys to the base of the bladder; 90s after contrast: combined corticomedullary-nephrographic phase scan through the whole abdomen; 10 min after contrast: a test image was captured at the level of mid-ureter and repeated twice, if needed, at 2 min intervals if no contrast was detected in ureters; 10–16 min after contrast: Excretory-phase scan from the top of kidneys to the bladder base							

^1.^ 3D TSE = Three Dimensional Turbo Spin Echo sequence

^2.^ FS = Fat Saturation

^3.^ Diffusion weighted echo planar imaging with automatically calculated ADC maps, FS and with five respective b factors (0, 200, 400, 600, and 800 s/mm^2^).

^4.^ TFE = Ultra-fast fat saturated unenhanced three-dimensional Turbo Field Echo (THRIVE)

* Overcontiguous acquisition slice thickness/ Reconstructed slice thickness

#### CTU

A triple-phase CTU protocol scheduled three hours after the MRU was conducted in accordance with the ESUR recommendations (Table 1). Furosemide was injected in a similar dose as in the MRU i.e. at 1–2 min before the injection of iodinated contrast (1.5 ml/kg, maximum dose of 100 ml) (Omnipaque 350 mgI/mL, Cork, Ireland) delivered via power injector at 4 ml/sec followed by 30 ml saline flush. Split contrast-boluses of 70 and 30 ml were used with the latter bolus ending 25 seconds before scanning. Axial reconstructions of 1mm and 3mm axial, coronal and sagittal images of all phases were saved into the digital archiving system which undertakes full option interactive multiplanar and volume reconstructions (Sectra PACS, version 15.1.20.2, Sectra Workstations IDS7, Linköping, Sweden). The duration of the examination was approximately 12–18 min.

### Image Interpretation

The CTU scans were interpreted independently and blindly by two radiologists (MP,SK) and those obtained from the MRU by two other radiologists (RV, MS). Each radiologist had over 20 years of experience in multimodality abdominal imaging. Investigations were evaluated for the presence of obstruction, tumors and additional findings. *A tumor* was defined as a solid lesion with contrast enhancement, *a benign stricture* as a smooth walled fixed narrowing without a distinguishable mass and *urothelial thickening* as an enhancing thickened segment with no definite discrete mass. Furthermore, the UUT was divided into six anatomical areas: upper pole cavities, middle and lower pole cavities, renal pelvis, and upper-, middle-, and lower ureter. For each anatomical area in each UUT, opacification at CTU and diagnostic visualization at MRU (for simplicity, both outcomes will be subsequently referred to as visualization) were scored into 6 categories: 1) 0%; 2) 1–25%; 3) 26–50%; 4) 51–75%; 5) 76–99% and 6) 100%. Furthermore, the distention of the UUT was reported in millimeters and measured with both CTU and MRU at three levels: a) the anteroposterior diameter of the renal pelvis, b) mid-ureter and c) distal ureter. The volume of the bladder was further assessed from CTU and the last MRU images by multiplying the longest 3-axis diameter measurements with a constant factor of 0.52 [14]. Two observers (MS, RV) evaluated side-by-side both CTU and MRU for UUT visualization. Furthermore, one observer (MS) assessed the visibility of UUT by additionally examining only the 5–10 min excretory sequences and these readings were compared to those obtained from the full examination.

Finally, the patients were asked to fill in a questionnaire rating each examination on a scale of 1–10 based on their own subjective impressions and to name their examination of choice. Patients were also asked if they would accept MRU as a possible sole follow-up examination.

### Radiation Dose

The effective dose of each patient was computed on the basis of scanned areas and individual scan protocol details using the CT-EXPO software (v.2.3.1., copyright Georg Stamm and Hans Dieter Nagel, Hannover/Buchholz, Germany). The consistency of the simulation dose parameters (CTDI, DLP) with the scanner patient dose report was verified.

### Statistics

The statistical analysis was performed with SPSS-software for Windows (version 19; SPSS, Chicago, Ill). Differences between groups were considered significant if the p-value was <0.05. Non-parametric Wilcoxon matched-pairs test was performed to compare CTU and MRU mean visualization scores as well as the mean differences between diameter measurements. A parametric paired t-test was used to compare differences between the mean volumes estimated by MRI and CT.

## Results

A total of 40 UUT units were evaluated. One obstructed nonfunctioning kidney was excluded only from the visualization score and distention analysis. The mean GFR was 77.7 (range 49–100). One excretory phase CTU was repeated 4 minutes after the first session which exhibited non-diagnostic opacification. The mean radiation dose was 9.2 mSv (range 5.8–18.0 mSv).

**Fig 2:**
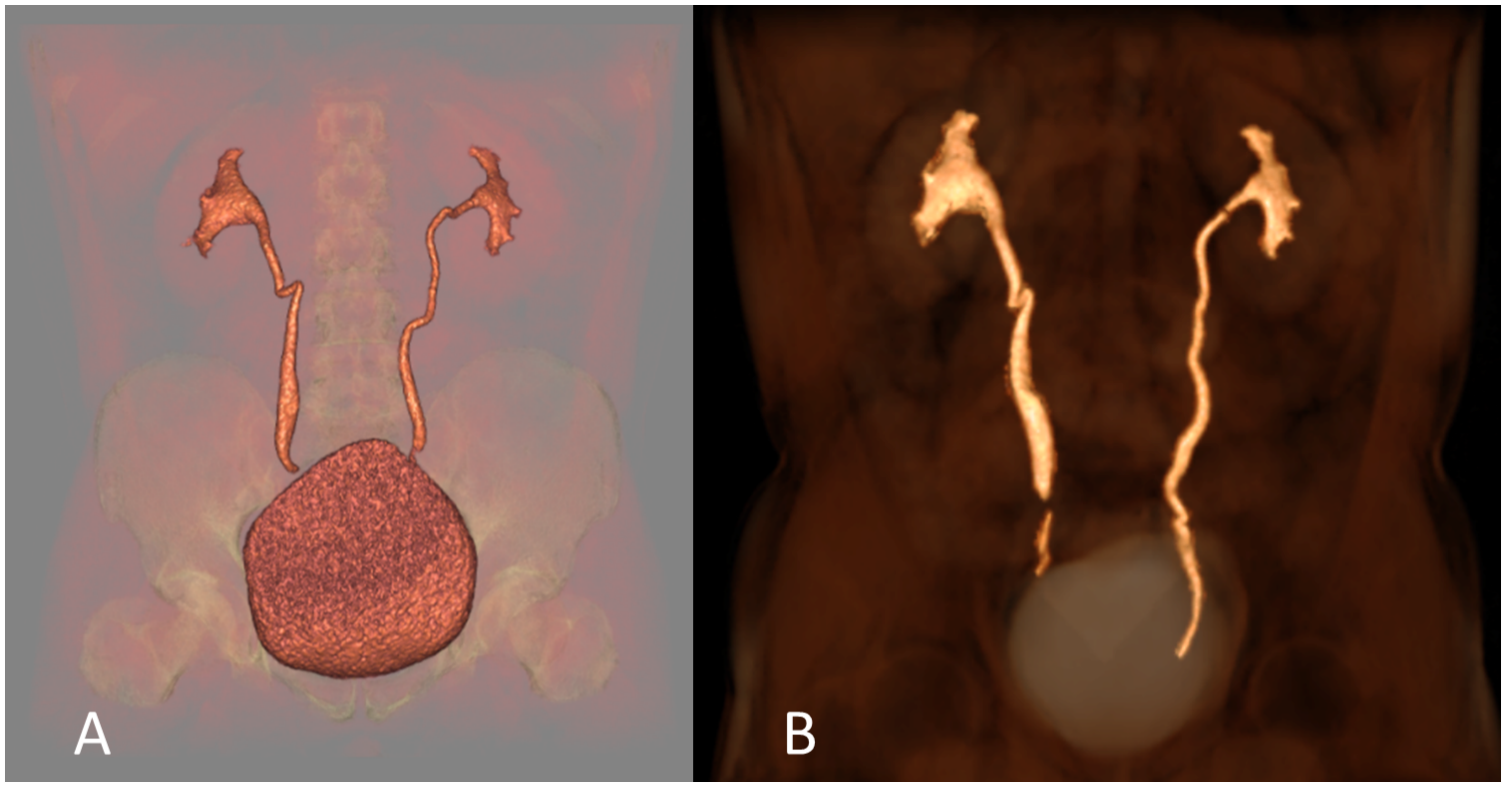
MR urography achieves comparable performance compared to CT urography. Three-dimensional Volume Rendering reconstruction of the urinary tract against a faded background from the images obtained with CT urography (A) and MR urography (B) excretory phases.MR urography achieved a comparable diagnostic performance.

**Fig 3:**
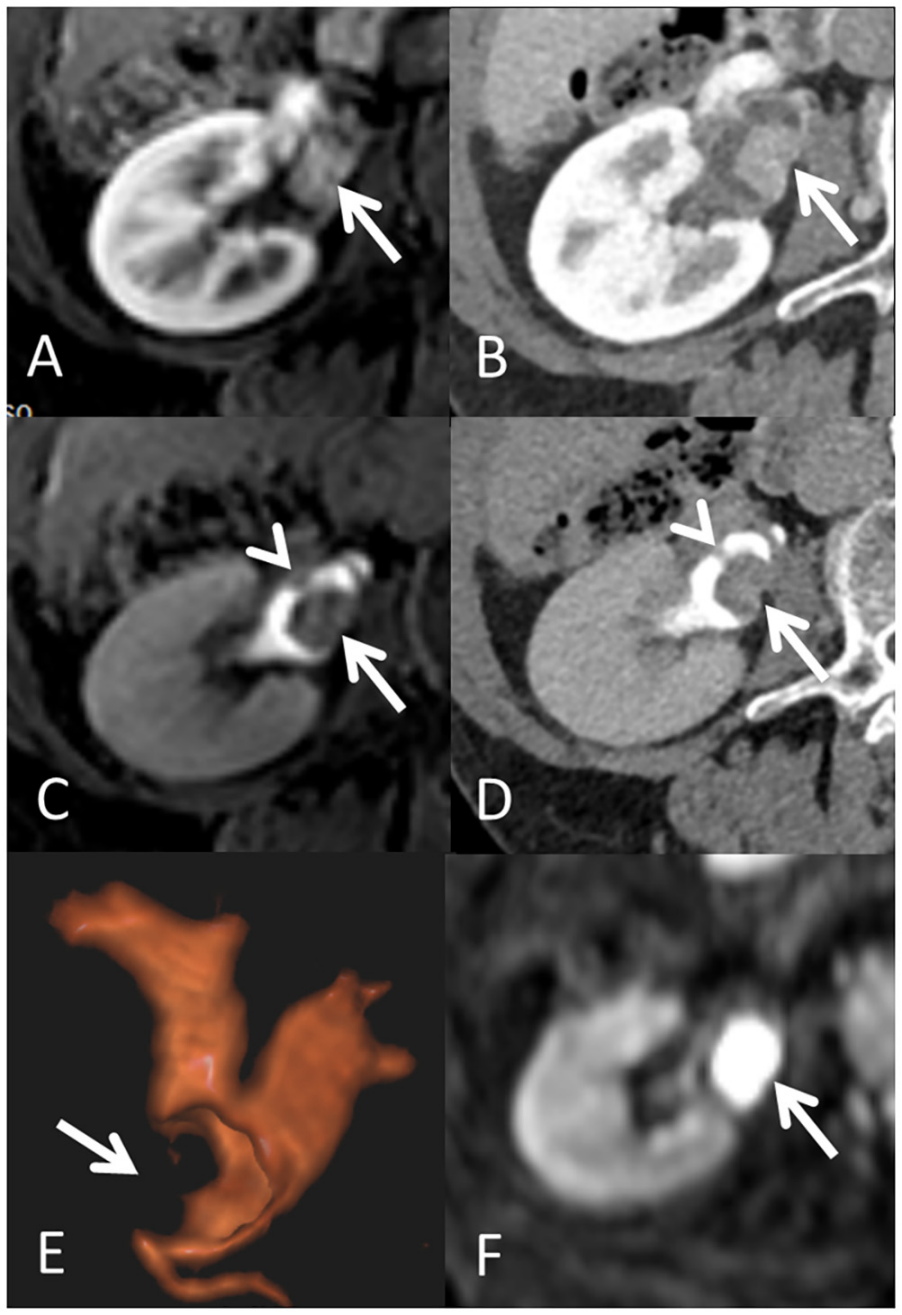
Visualisation of tumors at CT and MR urography. A 78 year old female patient presented with macroscopic hematuria. Axial contrast enhanced MRI (A) and CT (B) images at the level of the right renal pelvis showed an enhancing intraluminal mass (arrows) with no tumor extension outside the renal pelvis wall. A tumorous filling defect was also well visualized in the excretory phase MRU (C) and CTU (D) images and the presence of a small synchronous tumor on the opposite wall (arrowheads) was better recognized in the excretory phase images. The tumor filled the renal pelvic cavity (E, Arrow) resulting in subtotal occlusion with associated intrarenal-cavity dilatation as visualized on a postero-anterior three-dimensional volume rendering MRU. The tumor area showed restricted diffusion as estimated via the diffusion weighted imaging (F; b = 800; Arrow) with ADC values of 0.78 × 10^−3^ mm^2^/s (not shown). Final histopathology revealed a grade 2 pT1 urothelial carcinoma.

Both CTU and MRU achieved equivalent diagnostic performance (Fig 2) and both techniques detected two malignant tumors and one suspected ureteral tumor. The two confirmed malignant tumors showed restricted diffusion (Fig 3). Furthermore, an extraperitoneal tumor was diagnosed by both methods and a schwannoma was suggested by both MRU observers based on the tumor’s signal and DWI findings; this was confirmed at final histology. The benign etiologies of other obstructions (benign strictures and vascular compressions) were equally well visualized by both techniques. In patients investigated for dilatations, CTU showed five patients with grade 3 and two with grade 4 hydronephrosis. I One patient was found to have an indistinct small mass in the renal pelvic region at US. CTU and MRU both showed small benign septated cyst. At our hospital, the cost of MRU is approximately €626, compared with €235 for CTU.

**Fig 4:**
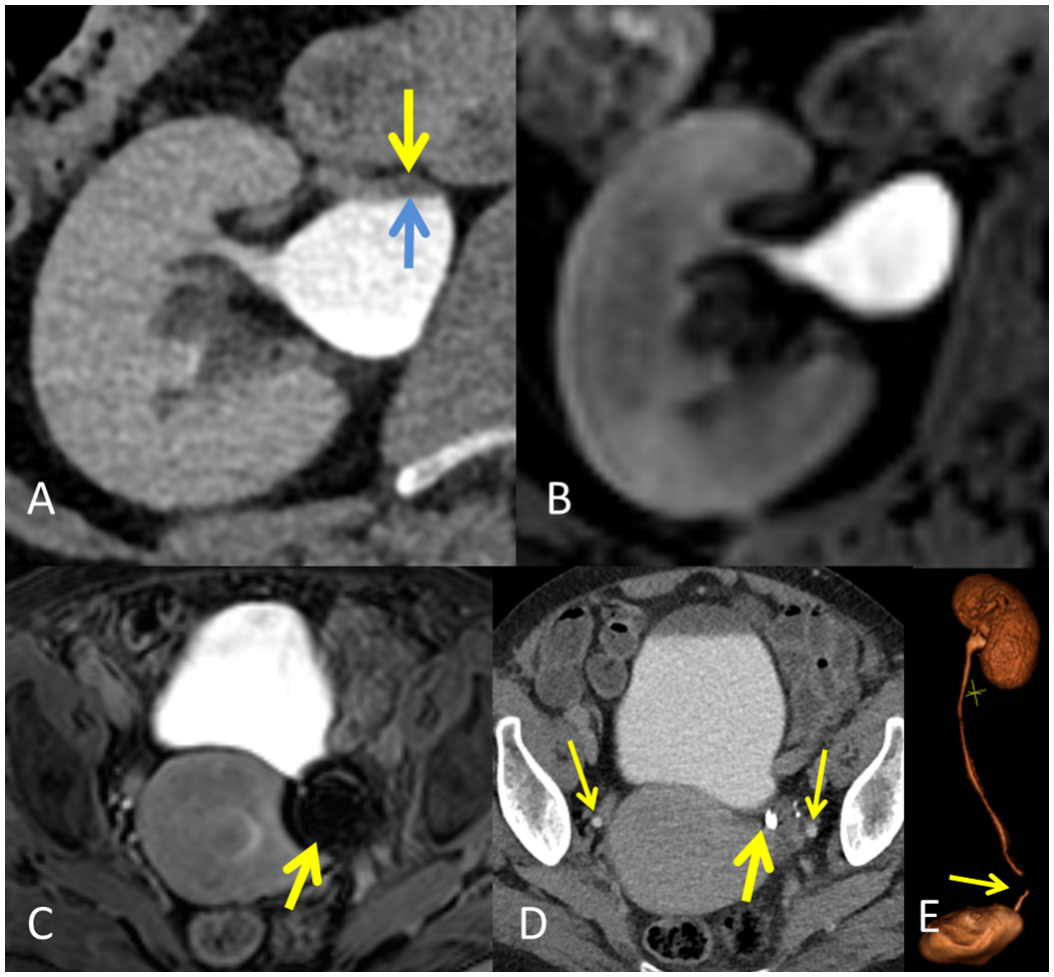
Artefacts encountered during imaging. The hydration protocol incorporated into the computed tomography resulted in better dilatation of the renal cavities as seen in image A (axial CT) compared to magnetic resonance excretory urography (MRU, image B), but occasionally at the expense of a contrast layering effect (Area between arrows). A susceptibility artefact due to the presence of a metallic sterilization clip in the MRU (image C, arrow) results in a void signal area. The clip produced no artefacts at CT (image D, thick arrow) and thin arrows show the position of distal ureters. The artefact at MRU impaired the visibility of a short ureteral segment as seen in the volume reconstruction MRU image E (arrow).

Visualization scores are presented in Table 2, and percentage visualizations in Table 3. Briefly, CTU showed slightly better intrarenal cavity visualization but consistently poorer visualization towards the distal ureters. MRU showed consistently slightly better visualization of the ureter. In the side-by-side analysis, MRU achieved better overall excretory phase visualization than CTU in 8/20 (40%) and equal performance in 10/20 (50%). One short segment of one distal ureter was not visualized at MRU due to the presence of a susceptibility artefact (Fig 4).

**Table 2:** Visualization scores.

Area	Comparison between methods												Comparison between readers					
	CT1	MR3	*p*	CT1	MR4	*p*	CT2	MR3	*p*	CT2	MR4	*p*	CT1	CT2	*p*	MR3	MR4	*p*
A	5.48	5.78	.014	5.48	5.73	.012	5.88	5.78	.046	5.88	5.73	.014	5.48	5.88	<.0001	5.78	5.73	.527
B	5.55	5.68	.225	5.55	5.73	.071	5.88	5.68	.005	5.88	5.73	.014	5.55	5.88	.001	5.68	5.73	.593
C	5.63	5.82	.052	5.63	5.73	.206	5.75	5.82	.317	5.75	5.73	.705	5.63	5.75	.025	5.82	5.73	.157
D	5.08	5.88	<.0001	5.08	5.75	.001	5.5	5.88	.066	5.5	5.75	.254	5.08	5.5	<.0001	5.88	5.75	.025
E	5.05	5.88	<.0001	5.05	5.75	<.0001	5.28	5.88	.011	5.28	5.75	.035	5.05	5.28	.019	5.88	5.75	.025
F	4.48	5.85	<.0001	4.48	5.78	<.0001	5.3	5.85	.001	5.3	5.78	.003	4.48	5.3	<.0001	5.85	5.78	0.18

Mean visualization scores of the upper urinary tract (UUT) as evaluated by four observers and their significance values in the comparison of the performance of computed CT urography and MR urography (whole examination at different time intervals jointly analyzed). Opacification at CT urography and diagnostic visualization at MR urography were scored into 6 categories: Score 1 = 0% visualization; 2 = 1–25%; 3 = 26–50%; 4 = 51–75%; 5 = 76–99% and 6 = 100% for each anatomical area (A-F) separately.

Areas: A = Upper cavities; B = Middle and Lower cavities; C = Renal pelvis; D = Ureter, proximal third; E = Ureter, middle third; F = Ureter, lower third. CT1 = CT first observer; CT2: CT second observer; MR3: MR third observer; MR4: MR fourth observer

**Table 3:** Percentage visualization of the upper urinary tract.

Area^#^	CTU Observer 1		CTU Observer 2		MRU Observer 3		MRU Observer 4	
	* V = 100% N (%)	V>75% N (%)	V = 100% N (%)	V>75% N (%)	V = 100% N (%)	V>75% N (%)	V = 100% N (%)	V>75% N (%)
A	25 (64.1)	37 (94.9)	39 (100)	39 (100)	35 (89.7)	39 (100)	33 (84.6)	39 (100)
B	27 (69.2)	38 (97.4)	39 (100)	39 (100)	31 (79.8)	39 (100)	33 (84.6)	39 (100)
C	31 (79.5)	38 (97.4)	35 (89.7)	38 (97.4)	37 (94.9)	39 (100)	33 (84.6)	39 (100)
D	21 (53.8)	34 (87.2)	35 (89.7)	35 (89.7)	39 (100)	39 (100)	34 (87.2)	39 (100)
E	19 (48.7)	34 (87.2)	31 (79.8)	33 (84.6)	39 (100)	39 (100)	34 (87.2)	39 (100)
F	14 (35.9)	23 (59.0)	25 (64.1)	35 (89.7)	38 (97.4)	39 (100)	35 (89.7)	39 (100)
Total	137 (58.5)	204 (87.2)	204 (87.2)	219 (93.6)	219 (93.6)	234 (100)	202 (86.3)	234 (100)

Percentage visualization (total = 100% and subtotal >75%) of different areas of the upper urinary tract (UUT) as visually evaluated by four independent observers with excretory phase CT and MR urography (whole examination with three time points jointly analyzed). Twenty patients with 39 UUT-units were evaluated.

*V = Excretory phase visualization of the urothelium.

^#^Area: A = upper cavities; B = middle and Lower cavities; C = Renal pelvis; D = Ureter, proximal third; E = Ureter, middle third; F = Ureter, lower third.

**Fig 5:**
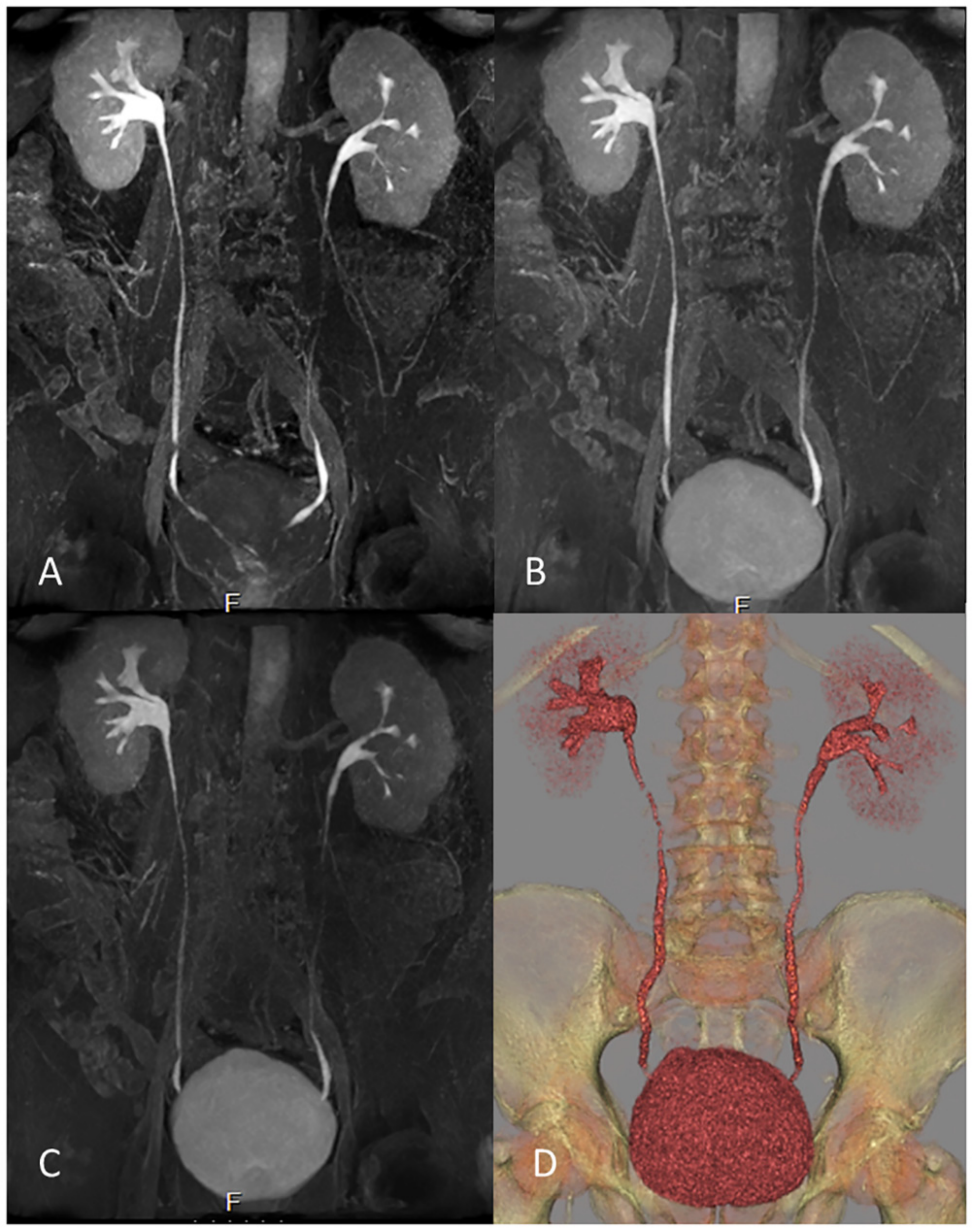
The value of MR imaging at different time intervals. MR urography maximum intensity projections at 5 min (A), 10 min (B) and 15 min intervals after the administration of contrast show no difference in visualization of the upper urinary tract (UUT) at MR-combined different time intervals. Different segments can be better visualized at different time intervals therefore improving the overall UUT visibility and provided comparable performance with CT urography (D, volume rendering reconstruction).

CTU rated the bladders as being more distended (mean 435.3 ml) than observed with MRU (mean 341.3 ml, p = 0.035). The renal pelvis was more distended at CTU (19.3 mm vs. 17.9 mm; p = 0.002), but no major differences were detected in ureteral distention (Table 4) (Fig 5). Four UUT-units (10.3%) showed layering effect at CTU (Fig 4). MRU visualization scores for the first 10 min showed consistently lower scores at all levels compared to those obtained in the full examination, but this reached statistical significance only at the level of middle and lower intrarenal cavities and mid-ureter (p<0.05).

**Table 4:** Diameter measurement of the renal pelvis and ureter.

Localization	MRU 5 min	MRU 10 min	MRU 15 min	CTU	P	P^1^	P^2^	P^3^
Pelvis (mm)	17.3	17.7	17.9	19.3	.180	.224	.085	.002
Proximal Ureter (mm)	5.5	5.8	6.1	6.3	.120	.091	.001	.299
Distal Ureter (mm)	5.2	5.4	5.4	5.0	.237	.854	.106	.060

The anteroposterior diameter of the renal pelvis as assessed at 5, 10 and 15 minutes in MR Urography (MRU) and the diameter of the proximal and the distal ureters obtained from MRU images as compared to those acquired with CT urography (CTU).

Abbreviations: P-value of statistical significance between MR urography (MRU) at 5 and 10 minutes; P^1^- value of significance between MRU at 10 and 15 minutes; P^2^- value of significance between MRU at 5 and 15 minutes; P^3^- value of significance between MRU at 15 minutes and CT urography (CTU).

Finally 16 patients (80%) chose CTU as their preferred examination (score CTU = 9.4 vs. MRU = 8.9), nevertheless all patients were willing to undergo MRU if it was deemed necessary as the only follow-up examination in the future due to its radiation-free profile.

## Discussion

Our results show that the performance of a comprehensive 3.0T-MRU protocol was equivalent to that obtained with CTU, generally considered as the gold standard. Furthermore, the visualization of the UUT, especially in the ureters, was more complete with MRU. This indicates that the 3.0T-MRU has the potential to achieve high accuracy in the evaluation of the UUT in high risk patients.

The distention of the intrarenal cavities was better with CTU due to the hydration and diuresis protocol used. Increased diuresis and consequently more filled bladder secondarily results in better distention and opacification of the calyces and pelvis [15]. Nevertheless this occasionally comes at the expense of a contrast layering effect which decreases opacification. Furthermore, full opacification of the slender and contractile ureters is still incomplete with one excretory phase CTU acquisition, whereas the possibility to conduct repeated excretory sequences at MRU proved useful in achieving full (i.e.100%) diagnostic visualization in up to 93.6% of all areas and >75% visualization in all patients.

Our results are in agreement with the previous report of Childs et al. [4], which showed that 3.0T-MRU is feasible and highly accurate, regardless of the frequent artifacts since these rarely interfere with the interpretations. We achieved >75% visualization of the UUT in all patients with multiple excretory sequences opposed to a corresponding value of 90.8% [4]. We also confirm that signal loss due to metallic susceptibility artefact impairs the visibility of UUT structures in close vicinity to these implants. Therefore this limitation of MRU should be acknowledged e.g. in the follow-up of patients after reconstructive urological surgery.

Until the present study, there has been no comparison in the same patient population of the actual performance of a comprehensive 3.0T MRU protocol with the triple-phase CTU approach. We found that the two procedures performed equally well which is in contrast to a previous retrospective study that applied a limited 1.5T-MRU protocol with only one T1-excretory sequence at 10 min as compared to split-bolus CTU [16]. Our study highlights that MRU should not try to duplicate CTU protocols, but since it is a safe modality, multiple excretory acquisitions are recommended since this can achieve better contrast visualization. Furthermore we stress the importance of utilizing a comprehensive protocol that includes also post-contrast enhancement phases e.g. the corticomedullary and/or nephrographic phase sequences in the complete evaluation of the UUT. There is recent evidence demonstrating the high accuracy of contrast enhanced CT compared to excretory phases [17], but it seems most likely that both phases complement each other [18]. Furthermore, based on previous CTU and MRU reports, it is apparent that single excretory phase imaging at 5 or 10 minutes results in incomplete UUT visualization, e.g. Dillman et al. [19] reported >75% opacifications of intrarenal cavities to be as low as 41%. Interestingly, in this study, the ureter was only slightly more distended at different MRU-time intervals and although this did not always reach statistical significance due to the small patient population, consistently better visualization was present at 15 min emphasizing the importance of repetition acquisitions at longer intervals after the induction of enhanced diuresis. Our excretory protocol was devised and refined years ago and it reflects the local expertise. We also emphasize that it is important to obtain images not only in the coronal plane but also in the axial orientation as this will facilitate the evaluation of possible small lesions or wall thickenings.

The accuracy of MRU in the evaluation of hematuria or high risk patients has yet to be determined. Nevertheless, an approximate sensitivity of 75% for UUT has been reported based on studies where most of the recruited patients have had relative contraindications to CTU, therefore rendering their results incomparable to normal CTU patient populations [6,20,21]. Furthermore, it might be also speculated that the thicker 3–4 mm MRU slice thicknesses used in those studies decreased the sensitivity.

MRU is not without its shortcomings; it is a rather lengthy examination which requires patient cooperation. Patient preferences are not usually reported in radiological comparative studies, but we believe that it is important to include the viewpoints of our patients in the evaluation process of new imaging modalities or protocols. Even though MRU was deemed to be slightly less patient friendly, all patients stated that they would be willing to undergo MRU again as the sole examination due to its safety profile. It is also true that the higher cost associated with the use of MRU is an issue of administrative concern in an era of cost containment. Equally important, quality of provided care should be assessed individually and whenever exposure to radiation or iodine-containing contrast material is considered an issue of concern, it is the responsibility of the treating physician and the radiologist to offer a suitable substitute, specifically MRU, without compromising quality. The availability of 3.0T MRI scanners and access are still limited compared to the more widely available 1.5T scanners. However, the availability of 3.0T MR scanners is continuously improving and gradually becoming more widely accessible. Furthermore, radiologists have increasingly gained experience in abdominal imaging at higher field strength.

Many patient undergo the high radiation dose investigations simply to rule out the rather rare possibility of UUT malignancy. The radiation dose of 9.2 mSv administered in our triple-phase MDCT protocol is within reported limits [1] but nonetheless is rather substantial; for comparison it exceeds 8 years of natural background radiation exposure in our country (on average 1.1 mSv/yr.). A crude estimation for the excess relative risk of death due to cancer from receiving a 9.2 mSv dose is one excess radiation induced death per 2000 exposed individuals if one applies the radiation-induced mortality rate of 5% per Sievert of radiation exposure [22]. MRU is therefore a reasonable alternative in patients who would be predicted to require repeated radiation examinations or in those with long life expectancies.

The small patient population is the major limitation of this study. Not all findings were operatively verified and additionally there were only a small number of tumors which is a further limitation

Although the role of diffusion weighted imaging (DWI) was not separately evaluated in this study, it is worth mentioning that DWI was most helpful in evaluating tumors in the present study and is a promising imaging modality for improving accuracy of staging and follow-up of urothelial cancers and adding further strength to MRU imaging [23].

To conclude, a comprehensive 3.0T-MRU protocol involving multiple contrast-enhanced and excretory sequences has the potential to display as high accuracy as can be achieved with CTU while eliminating the risk of exposing the patient to ionizing radiation.
